# Community-Based Efforts to Reduce Violence: A Scoping Review on the Implementation of Cure Violence

**DOI:** 10.1177/00469580251360956

**Published:** 2025-07-28

**Authors:** Sara Solomon, Caterina G. Roman, Melissa Davey-Rothwell, Ruth Abaya, Daniel Webster, Shannon Frattorolli

**Affiliations:** 1 University of Pennsylvania, Philadelphia, PA, USA; 2 Temple University, Philadelphia, PA, USA; 3 Johns Hopkins Bloomberg School of Public Health, Baltimore, MD, USA

**Keywords:** implementation science, gun violence, scoping review, evidence-based practice

## Abstract

This scoping review aimed to identify implementation determinants, strategies, mechanisms, and outcomes of Cure Violence (CV) programs to develop an Implementation Research Logic Model (IRLM) and integrate Implementation Science (IS) principles into community violence intervention research and practice. Following Mak and Thomas methodology, we conducted a comprehensive literature search across multiple databases, including peer-reviewed articles and grey literature from 2008 to 2023. Data extraction focused on implementation constructs, study characteristics, and CV intervention details. Thematic analysis was used to synthesize findings and develop the IRLM. The review included 29 publications covering 19 distinct CV programs, primarily in the United States. While 62% of studies incorporated IS elements, only one explicitly mentioned using an IS framework. We identified 42 implementation strategies across 6 categories: hiring and retention, training, violence interruption and detection, identifying and treating high-risk individuals, changing community norms, and general implementation and sustainability. Key contextual determinants influencing implementation included funding stability, community trust, and staff characteristics. The adapted IRLM visually depicts the relationships between implementation strategies, mechanisms, and outcomes. This review highlights the need for greater integration of IS principles in CV research and practice. The proposed IRLM provides a framework for practitioners to guide implementation and for researchers to design more rigorous studies unpacking the layers of implementation, including strategies, mechanisms, and outcomes that contribute to the variation in the effectiveness of CV across different contexts. Future research should focus on testing specific implementation strategies and measuring a broader range of implementation outcomes beyond fidelity.

## Introduction

Cure Violence is a community violence intervention (CVI) that employs frontline staff who are credible messengers due to their prior involvement in gangs or street groups. These staff members work to interrupt violence, connect individuals to resources, and change community norms.^1,2^ Cure Violence (CV) has been replicated and evaluated in at least 12 communities globally, including urban areas in the United States (n = 6), Canada (n = 1), Latin America (n = 3), and the Caribbean (n = 2).^3^ Since the initial evaluation demonstrated effectiveness in 4 of 7 neighborhoods,^4^ consecutive studies have shown partial or no effectiveness in reducing at least one measure of violence.^4-8^ Some peer-reviewed studies and reports have even shown evidence of violence-generating effects.^6,9-11^ As a result, the authors conclude that CV is “promising” but “not proven effective.”^12^

The mixed outcomes highlight the need to understand whether failures stem from implementation issues or weaknesses in the intervention model. For example, why does CV work in some neighborhoods but increase violence in others? Are certain CV strategies more effective than others? Do outcomes depend on implementation fidelity versus the context in which implementation occurs? With limited resources, where does a community focus its efforts?

Understanding CV through the lens of implementation science (IS) may help answer these types of questions. While traditional effectiveness studies examine *whether* an intervention works, IS research seeks to understand *how and why* the intervention works (or fails to produce an effect) in a particular context.^13^ IS encompasses research that evaluates the process of implementing evidence-based interventions,^14,15^ as well as hybrid designs that examine both the effectiveness of a promising intervention and its implementation.^16^

In the field of IS, implementation strategies are “methods or techniques used to enhance the adoption, implementation, and sustainability of a program or practice.”^17^ Defining and reporting these strategies are paramount for intervention fidelity and uptake, as well as tailoring and adaptation across different settings and contexts.^17^ Defining strategies may also serve as a template for more rigorous study designs to test and understand which strategies are linked to positive outcomes and which are not. Identifying strategies also helps understand potential mechanisms by which the intervention initiates results.

The implementation research logic model (IRLM) is a tool that visually depicts implementation strategies directly related to the factors that hinder or help implementation. The IRLM links strategies to the mechanisms of action that affect proximal (eg, implementation) and distal outcomes (eg, effectiveness). Through these components, the IRLM illuminates how variation in intervention delivery and context can be assessed to maximize the impact of an intervention.^18^ Compared with a traditional logic model,^19^ the IRLM focuses on the implementation process and mechanisms between an intervention and its outcomes. Furthermore, IRLM displays both effectiveness and implementation outcomes, such as acceptability, appropriateness, cost, fidelity, penetration, and sustainability.^13,20^

To our knowledge, an IRLM does not exist for CV. While the core components and related activities are documented,^2^ no conceptual framework captures the strategies or mechanisms that impact outcomes. One scientific review (2015) provides a theoretical basis and traditional logic model that supports implementation^1^ but does not capture the extent of implementation in a way that may help explain variations in effectiveness. This represents a knowledge gap and a missed opportunity to apply a framework to optimize implementation strategies and understand the mechanisms by which they achieve desired outcomes.

We conducted a scoping review to address this gap and identify how practitioners and researchers conceptualize implementation determinants, strategies, mechanisms, and outcomes in the peer-reviewed and gray literature. Our primary research questions were driven by the need to guide a local adaptation of CV and included: 1) How do practitioners and researchers define and conceptualize implementation determinants, strategies, mechanisms, and outcomes? 2) What are the key gaps in the literature? By answering these questions, we aimed to propose an IRLM that could inform, guide, and help practitioners, policymakers, and researchers develop more effective Cure Violence (CV) interventions, incorporate rigorous implementation science (IS) designs, and ultimately facilitate the integration of IS into the broader ecosystem of community violence intervention (CVI) research and practice.

## Methods

We undertook a scoping review to explore the literature comprehensively, map the breadth of evidence available on the topic, and identify key concepts, gaps in the research, and the types of evidence available. This approach is ideal for topics for which existing research is heterogeneous or poorly established. We followed the Mak and Thomas (2022) scoping review methodology, which provides researchers with step-by-step guidance.^21^ We relied on the Preferred Reporting Items for Systematic Reviews and Meta-Analyses extension for Scoping Reviews (PRISMA-ScR), which identifies the 20 essential reporting items via a checklist to guide the reporting of findings^22^ (available as supplementary materials), and registered the protocol in December 2023 (DOI 10.17605/OSF.IO/GYXQ2).^23^

### Step 1: Identifying the Research Questions

The research questions were inspired by a Philadelphia community planning to implement a CV intervention. They were developed under the guidance of the lead author with input from implementation scientists, violence prevention researchers, and practitioners. Our primary objective was to understand how practitioners and researchers define and conceptualize CV implementation determinants, strategies, mechanisms, and outcomes. We also examined gaps in the literature and areas where further investigation and application of IS principles are needed.

### Steps 2 and 3: Identifying and Selecting Studies

We included peer-reviewed journal articles, reports, dissertations, and briefs/summaries in English from 2008 to 2023 that reported on any aspect of effectiveness or implementation related to Cure Violence (formerly called Ceasefire). Articles were excluded if they did not focus explicitly on Cure Violence (CV) (eg, focused deterrence programs), were not in the timeframe, or if they were conference abstracts, simulation studies, systematic reviews, opinions, or critical discourse papers.

We developed initial search terms, including “Cure Violence” OR “Ceasefire” AND “violence,” to capture publications related to CV. Given the initial results, we further refined the search terms with “NOT war” to exclude unrelated studies. We then combined the search results with search terms related to the core CV components: “street outreach,” “credible messengers,” and “violence interruption.” Given the focus on IS, we added keywords relevant to the field, including determinants, facilitators, barriers, mechanisms, outcomes, outputs, processes, and impacts. We imported them into Covidence,^24^ a systematic and scoping review web-based management platform. And screened titles from PubMed, Scopus, Google Scholar, Catalyst JHU, and EBSCO Criminal Justice Abstracts with Full Text. As a final step, we integrated citations from prior reviews and reports.^3^

A primary and secondary reviewer screened the titles and abstracts of each paper and checked the agreement, discussed discrepancies, and restarted screening until consensus was achieved. Next, the primary author (who was also a coder) used the inclusion and exclusion criteria to conduct an independent, full-text review for the final selection of articles.

### Step 4: Charting the Data

We developed a charting system based on an existing codebook^25^ to capture IS components and collect information on implementation determinants, strategies, mechanisms, and outcomes. We used terms and definitions from the IS literature to guide our extraction.^17,26-29^ We focused on implementation constructs from the IRLM^18^ while collecting information on the study design and CV intervention, including funding, staffing, size, and location. To define implementation strategies, we utilized the Expert Recommendations for Implementing Change (ERIC) database, which provides a taxonomy and standard terminology for implementation strategies.^17^ We subsequently employed Proctor’s definition for specifying and reporting implementation strategies (2013) to define the operationalization of each strategy.^17^ We classified implementation outcomes via Proctor’s framework (eg, acceptability, adoption, appropriateness, costs, feasibility, fidelity, penetration, and sustainability).^20^ Finally, we organized the IS constructs by CV components (eg, violence interruption and detection, community mobilization), drawing from our literature review and professional expertise. This allowed for a systematic approach to understanding how IS principles apply to specific aspects of the CV model.

### Step 5: Collating, summarizing, and reporting the results

After completing the data extraction, we conducted numeric and thematic analyses to identify the most salient aspects of the review. Numeric analysis described intervention characteristics and the type and extent of implementation research employed. For thematic analyses, the primary reviewer coded the data with input from the study team. These included the factors in Table 1, such as the study design and factors influencing implementation. We employed a pragmatic approach, balancing deductive—preexisting concepts derived from the literature and conceptual models—and inductive open coding procedures, allowing for greater interpretation driven by the data.^30^ Throughout, the reviewer practiced reflexivity by journaling to capture thoughts that arose from examining and interpreting the data.

**Table 1.** Description of Publications in Scoping Review.

Program name	City, state	Study author (year)	Pub type	Abbreviated study aim	Study design	Evidence of effectiveness*	IS outcomes (Proctor, 2011)	Other Implementation Reported	Factors influencing implementation** ⊄
Aim4Peace Violence Prevention Program	Kansas City, MO	Stewart (2021)	Peer-reviewed journal	Examine conflict mediation and resolution strategies within context of broader model.	Case report	Not measured	None	Strategies: Conflict mediation	(+) Trust fostering a sense of support and equal power among involved parties; Violence interrupters and outreach workers modeled reformed behavior and understanding of lifestyle; Substantial engagement from youth in research activities
		Watson-Thompson (2022)	Report	Examine process and effectiveness of reducing gun violence from 2018 to 2020	Mixed Methods	Mixed	None	Characteristics of Implementation process	(+) Constant community presence, trusted relationships with community members (−) Funding reductions
Cure Violence (Formerly Ceasefire)	Chicago, IL	Skogan (2008)	Report	Evaluate process and impact on reducing gun violence	Quasi-Experimental, Mixed Methods	Yes	None	Characteristics of Implementation process Determinants: Successes and challenges	(+) Hiring panel for impartiality, multi-year grants ensured stability, strong support from host agencies with resources (−) Agencies with conflicting agendas, high staff turnover, unstable funding, program limited to small areas
		Dymnicki (2013)	Peer-reviewed journal	Understand characteristics associated with successful and unsuccessful violence interruption.	Qualitative research	Not measured	None	Determinants: facilitators and barriers (f/b)	(±) Severity of violence; level of control felt by worker; Responsiveness of involved parties; Existing relationships with client or family; (−) Complications from existing relationships with rival groups; Ongoing conflicts among known parties
		Gorman-Smith (2014)	Report	Determine community and client attitudes and perceptions on impact of reducing neighborhood crime and violence	Qualitative research	Not measured	None	Mechanisms: Awareness of Intervention	(−) Constrained resources, limited engagement with high-risk youth
		Slutkin (2014)	Report	Provide an overview of original model and recommendations for adaption	Case report	Yes	None	Characteristics: Implementation process	(+) Adaptable model with local partnerships
Ceasefire New Orleans	New Orleans, Louisiana	City of New Orleans (2016)	Report	Evaluate process and impact on reducing gun violence	Mixed Methods	Yes	None	Characteristics: Implementation process	(+) Early childhood development focus, targeted smaller violent groups (−) External socioeconomic factors affecting violence, educational and healthcare barriers
		McVey (2014)	Peer-reviewed journal	Evaluate the effectiveness in reducing penetrating trauma rates	Quasi-Experimental	No	None	None	(+) Coordination with community and police-based programs, regular quality improvement meetings, ongoing data collection (−) Limited data on homicide victims outside hospital
Crisis Management System (CMS)	New York, New York	Butts (2015)	Report	Evaluate impact on reducing gun violence	Quasi-Experimental	Mixed	None	None	(+) Community-led approach, identification of high-risk individuals (−) Lack of details on sustainability and program adaptations
Cure the Streets	Washington DC	Chwalisz (2023)	Peer-reviewed journal	Evaluate impact on reducing gun violence	Quasi-Experimental	No	None	None	(+) High resource infusion (−) COVID-19 disruptions
Does not specify	San Pedro Sula, Honduras	Ransford (2016)	Report	Report on adaptations and phases of implementation	Case report	No	Feasibility	None	(+) Phased implementation to improve safety, credibility, and community relationships (−) Limited resources
Man Up Inc, GMACC, Bronx Connect, SAVE, BRAG North, BRAG Northwest	New York, New York	DeFries Gallagher (2021)	Dissertation	Gain staff insight into the recommended implementation and replication of model	Qualitative research	Not measured	None	Characteristics: Implementation process	(+) Consistent messaging, high access to wraparound services for participants (−) Job danger, staff turnover, burnout, unsustainable funding
One Vision One Life	Pittsburgh, PA	Wilson (2010)	Report	Assess the extent of program implementation and impact on community violence	Quasi-Experimental, Mixed Methods	No	Fidelity	Transferability, Adaptation, Dosage; Determinants: f/b	(+) Comprehensive low-cost model with strong community relations (−) Complexity in multi-agency coordination, low funding limited impact on broader social factors
Operation Ceasefire Newark	Newark, New Jersey	Boyle (2010)	Peer-reviewed journal	Examine the impact on gunshot wound admissions	Quasi-Experimental	No	Fidelity	None	(+) Focused on high-risk individuals in areas with high gun violence (−) Unclear sustainability
Philadelphia Ceasefire	Philadelphia, PA	Roman (2017)	Report	Examine the impact of the program on aggregate-level shootings	Quasi-Experimental	Yes	None	None	(+) Well-trained staff, use of Violence Interrupters (VIs) and Outreach Workers (OWs), strong community mobilization (+/−) Dependency on community and political support
Project REASON	Trinidad and Tobago, West Indies	Maguire (2018)	Report	Evaluate the extent of implementation, impact on outcomes, and cost-effectiveness	Quasi-Experimental	Mixed	Fidelity, Cost	Determinants: f/b	(+) Adaptability to local context, inter-agency collaboration, cost-effective (−) Complexity, lack of partnerships with social services, staffing issues
		Adams (2023)	Peer-reviewed journal	Analyze implementation process and challenges	Qualitative research	Other - Decreased community perceptions of crime	None	Characteristics: Implementation process; Determinants: barriers	(+) Strong partnerships with police, government, and health organizations (−) Insufficient staffing limited engagement and support, lack of resources for participant outreach activities
Safe Streets and Cure Violence (formerly Ceasefire)	Baltimore, MD and Chicago, IL	Whitehill (2011)	Dissertation	Describe the tactics outreach staff utilize to prevent shootings and identify implementation practices that contribute to an effective street outreach program.	Mixed methods, Case report	Yes - conflict resolutions	Fidelity	None	(+) Direct oversight allowed flexibility, strong relationships with participants and street organizations (−) Staff overextension, lack of resources for participant needs, difficulty mediating conflicts with large sums involved
Safe Streets	Baltimore, MD	Webster (2009)	Report	Describe program implementation and most effectiveness strategies; estimate effects on attitudes, norms and severe violence	Quasi-Experimental, Mixed Methods	Mixed	None	None	(+) Targeted high-risk youth, effective OW and VI relationships with clients (−) Inconsistent implementation leading to variable outcomes
		Webster (2012)	Report	Evaluate implementation, community attitudes toward gun violence and impact on shootings	Quasi-Experimental, Mixed Methods	Mixed	None	None	(+) Program implemented in high-need areas (−) Varied attitudes toward gun violence among neighborhoods, staff retention challenges
		Whitehill (2013)	Peer-reviewed journal	Describe characteristics of conflict mediations and associations between successful conflict mediations and associated risk factors	Cross Sectional	Not measured	None	Strategies: conflict mediations	(+) Effective in gang-related conflict mediation (−) More difficult to mediate conflicts involving weapons and retaliatory motivations
		Webster (2013)	Peer-reviewed journal	Examine program and impact on homicide and nonfatal shootings	Quasi-Experimental	Mixed	Fidelity	None	(+) Fidelity to program implementation, effective conflict mediation (−) Weak neighborhood organizational support
		Whitehill (2014)	Peer-reviewed journal	Examine key components, processes and variations of street conflict mediation	Multiple Case Study	Mixed	None	Characteristics: Implementation process; Strategies: street conflict mediation; Determinants: f/b	(+) Focus on building trust and respect in mediation (−) Retaliatory conflicts involving money hardest to mediate
		Milam (2016)	Peer-reviewed journal	Examine perceived norms and attitudes on gun violence pre- and post-implementation	Mixed Methods	Other - Improved attitudes towards gun violence	Acceptability	Exposure; Attitudes toward gun violence	(+) Positive change in community attitudes toward violence
		Buggs (2022)	Peer-reviewed journal	Evaluate program impact and examine how civil unrest and increases in community violence influence program outcomes	Quasi-Experimental	Mixed	None	None	(+) Initial funding boost due to positive outcomes (−) Funding cuts in low-performing areas, evaluation comparisons difficult
Save Our Streets (SOS)	Brooklyn, NY	Picard-Fritsche (2013)	Report	Evaluate process and impact on reducing gun violence	Quasi-Experimental	Yes	Fidelity	Transferability	(+) Visibility builds community trust, flexible roles combining outreach and violence interruption (-) Racial tensions, limited funding, reliance on anecdotal data for measuring success, retention challenges
Save Our Streets (SOS) and Man Up!	New York, NY	Butts (2017)	Report	Test relationship on social norms and examine relationships between norms and confidence in police	Survey research	Other: Increased confidence in law enforcement	None	Attitudes toward implementation	(+) potential positive impact on police legitimacy and confidence in law enforcement. (-) Unclear causality between violence reduction and police legitimacy
	Bronx, NY	Delgado (2017)	Report	Evaluate effectiveness on reducing neighborhood violence	Quasi-Experimental, Mixed Methods	Yes	Fidelity	Participant characteristics and implementation process	(+) Flexibility in approach, low cost, additional services supported by external funding (−) High complexity, difficulty in coordinating with other agencies
TRUCE Project	Phoenix, AZ	Fox (2015)	Peer-reviewed journal	Evaluate process and impact on reducing gun violence	Quasi-Experimental, Mixed Methods	Mixed	Fidelity, Penetration	Participant and strategy characteristics	(+) OWs and VIs with relevant backgrounds, flexible and adaptable implementation (-) Challenges with community engagement, difficulty replicating in lower-density areas

* Yes refers to studies that conclude the model is effective. No refers to studies that conclude the model is effective or has negative outcomes. Mixed refers to studies that conclude the model was effective in some neighborhoods/units but not effective or iatrogenic in others.

** (+) indicates facilitators/ factors that help implementation; (−) indicates barriers/challenges to implementation; (±) indicates either a facilitator or barrier depending on context/situation; ⊄ indicates not a complete list.

To build the IRLM, we worked through an iterative process using the results from this review and existing IS constructs and CV conceptual models.^1,2^ We adapted a template by Smith et al (2020), which reports on contextual determinants, strategies, potential mechanisms, and implementation and effectiveness outcomes. We chose the IRLM because of its focus on clearly defining implementation strategies used to promote implementation, such as the uptake, acceptability, and adoption of the intervention.^15^ We organized the IRLM based on the CV components listed on the CV Global website. Given that the core components did not fully capture the implementation scope, we added categories on hiring and retention, training, and sustainability to facilitate practical application.

Selecting strategies for the IRLM involved a multistep process. First, we used data from the scoping review to develop a list of strategies and then grouped and refined them based on commonalities across studies. A strategy was considered common if it appeared in more than one paper. Next, we matched strategies to the ERIC database, standardizing terminology. We then categorized strategies from start-up/early implementation to long-term sustainability, providing a comprehensive overview (Table 2). Finally, we synthesized the data to create a condensed version for the IRLM and cross-checked strategies with contextual determinants to ensure we considered all relevant factors.

**Table 2.** Cure Violence Implementation Strategies (n = 42).

Domain*	Strategy Name**	Strategy Definition
Staff Hiring and Retention ^1-5^ (n = 5, 17%)	Create a hiring panel	Incorporate a formal decision process for hiring with a panel of 5-7 community stakeholders (ie, clergy, residents, business owners, law enforcement).
	Obtain background checks	Perform formal or informal background checks to ensure workers are not currently involved in illegal activities or have prior offences against women or children.
	Conduct local consensus discussions**	Engage team members in weekly team meetings to conduct local consensus about workflows and adaptations to program.
	Tailor employee support and supervision	Provide tailored support and supervision in administration, HR, operations, and professional training, including computer skills and guidelines on professional skills.
	Promote employee wellness	Provide wellness support to staff, including scheduled days off, wellness activities to address burnout, and access to trauma-informed services.
Training and Technical Assistance^1,5-8^ (n = 6, 21%)	Conduct initial training	Conduct initial training on conflict mediation, case management, trauma-informed care as well as procedures for record keeping and data entry and management.
	Conduct community trainings	Conduct trainings for community members on topics such as finishing school, gaining employment, problem-solving, decision-making, anger, alternatives to violence, and nonviolent communication.
	Audit and provide feedback**	Conduct weekly meetings and observations with staff to ensure fidelity, troubleshoot issues and build skills.
	Conducting ongoing training**	Conduct follow-up trainings on topics such as promoting behavior and norm changes, active listening, suicide prevention, and motivational interviewing, as well as resume building and other professional skills.
	Shadow other experts**	Shadow expert outreach workers and violence interrupters in the field.
	Create safety measures	Create a safety protocol prior to conducting outreach and interruption activities, including travelling in pairs, reflective clothing, information gathering before a mediation, and walking away from situations.
Violence Interruption and Detection ^1,4,6,8-23^ (n = 10, 34%)	Appeal to both parties	Orchestrate a solution that is appealing to both parties.
	Build trusting relationships	Workers build relationships with community leaders and high-risk potential participants prior to conflict mediation.
	Communicate pettiness and consequences	Workers emphasize factors such as going to jail, getting wounded or killed, impacting parents, children, siblings. Worker may share personal experiences with being in jail and other negative consequences.
	De-escalate the situation	De-escalate the intense situation by calming participants down, taking participants away from the scene, and contacting family, friends or community leaders
	Enlist community volunteers	Enlist and train community volunteers to capture local knowledge and help stop a conflict as it is occurring or call the CV worker.
	Isolate	Isolate high-risk individuals by keeping to themselves, staying in their home or block, or leaving the neighborhood altogether. Separate the potential shooter from the intended victim or group.
	Negotiate conflict	Negotiate differences between gang members or other potentially violent individuals. After an agreement is reached, both parties verbally agree.
	Tailor strategies**	Tailor strategies based on the conflict at hand.
	Use social media	Workers use social media to follow high-risk participants to keep tabs on any potential conflicts.
	Use a stepwise approach	Respond to conflict by: (1) assess and manage immediate threats, (2) decide how to structure their intervention (I e bringing people together, acting as a go-between, using a relevant 3rd party), (3) use specific reasoning and persuasion tactics, (4) broker a resolution and agreements.
Identifying and treating individuals at high risk of violence^3,4,6,8,10-12,14,16,18,20,24,25^ (n = 8, 28%)	Conduct risk assessment	Assess potential participants to determine risk based on: recently released from prison, recently shot, active in a violent street organization or gang, involved in high-risk street activity (ie, selling drugs), weapon carrier (automatically high risk), close to a person of a recent shooting.
	Create an individualized plan	Create client-identified goals (ie, going back in school, GED programs, employment, substance abuse or mental health treatment programs, parenting classes, anger management).
	Practice active listening	Listen to the needs of the high-risk youth without judgement or criticism.
	Promote network weaving**	Establish connections with a range of service providers and community partners to offer referrals for social, legal, and health services (eg, obtaining birth certificates, social service cards, finding housing) and support lifestyle changes (eg, leaving a gang, joining sports or recreational activities).
	Build trusted relationships	Maintain a consistent presence in order to build trust and develop relationships, contacting individuals at least weekly.
	Involve family members**	Consider the involvement family members and mentors in stages of the case management process.
	Assist with employment	Outreach workers and case managers support every step of the job-seeking process, including resume writing, filling out applications, attending job fairs, securing appropriate attire, conducting mock interviews, and providing transportation to interviews.
	Engage with critical partners	Engage with critical partners such as hospitals, schools, and law enforcement to best identify and treat highest risk participants
Changing community norms^1-4,6,8-13,15-18,20,24,26^ (n = 6, 21%)	Build a coalition	Build a coalition/advisory board of local collaborators with regular meetings to provide public input and help link site activities (ie, Coalition members serve on hiring panels, display materials, refer participants, host jobs, support events)
	Canvas the community consistently	Canvas the target neighborhood during daytime and nighttime hours consistently, talking to people, offering educational materials, and services for grief support, safety, and individual and community healing.
	Conduct shooting response	Conduct shooting responses within 48 to 72 hours to show support for the community, gather information, and make connections with victims, family members, and friends in efforts to prevent retaliatory violence.
	Develop tools for quality monitoring	Develop, test, and introduce quality-monitoring systems that are specific to the strategies being implemented
	Develop and distribute educational materials**	Develop and distribute educational materials that provide information on how to decrease shootings and promote non-violent conflict resolution strategies; this includes social media.
	Inform and engage local opinion leaders**	Inform and engage local leaders, such as clergy and schools, by participating in local events to establish credibility, create positive engagement opportunities, and build relationships that foster trust and promote adoption.
General implementation + Sustainability^1,3,6-8,10,14,16-18,20,24,26^ (n = 7, 25%)	Access new funding**	Access long-term funding to expand and sustain staff and programming.
	Create an implementation blueprint**	Create a blueprint for implementation along with implementation checklists for clarity of goals and procedures.
	Obtain formal commitments**	Obtain formal commitments with strategic stakeholders including law enforcement, schools and hospitals.
	Phase implementation	Phase implementation efforts by starting with planning and community engagement and gradually move to full implementation
	Provide feedback on reporting	Supervisors provide feedback on data reporting to help ensure high-quality data for future research and monitoring
	Identify and prepare champions**	Identify local opinion leaders including leaders of street organizations, school principals, politicians, and faith-based leaders.
	Use data**	Analyze data on an ongoing basis to identify and adapt target areas and track changes in violent activity over time.

*Note.* See References Whitehill et al (2014)^8^; Wilson (2010)^9^; Fox et al (2015)^10^; Watson-Thompson et al (2022)^31^; Slutkin et al (2014)^32^; City of New Orleans (2016)^33^; Gallagher (2021)^34^; Adams and Maguire (2023)^35^; Dymnicki (2013)^36^; Maguire and Oakley (2018)^37^; Stewart et al (2021)^38^; Whitehill (2011)^39^; Gorman-Smith and Cosey-Gay (2006)^40^; Butts et al (2015)^41^; Milam et al (2016)^42^; Picard-Fritsche and Cerniglia (2013)^43^; Delgado SA et al (2023)^44^; Ransford (2016)^45^; Powell et al (2015)^48^; Chwalisz (2023)^49^; Roman et al (2017)^50^; Webster (2018)^51^; Whitehill et al (2015)^52^; Wesley et al (2009)^53^

* The number refers to the number of studies we extracted data from relevant to the domain and the percentage (rounded) based on the total amount of studies reviewed.

** Indicates an ERIC strategy (n = 14, 33%), retrieved from the Expert Recommendations for Implementing Change (2015).^49^

To identify potential mechanisms and outcomes in the IRLM, we used data extracted from the scoping review, as well as the CV logic model proposed by Butts et al (2015).^1^ The implementation outcomes were further guided by Proctor’s framework and the Consolidated Framework for Implementation Research (CFIR).^20,26^

## Results and Synthesis

### Article Search

The search identified 123 documents that were imported into Covidence^24^ for review. After 29 duplicates were eliminated, 94 documents remained for screening. Among these, 46 publications that did not meet the inclusion criteria were excluded, and the titles and abstracts of the remaining 48 publications were reviewed. The level of agreement between the 2 reviewers at this stage was 0.89. We further excluded 19 documents for not having an explicit focus on CV (n = 10), being conference proceedings (n = 4), not discussing the implementation or effectiveness of CV (n = 3), being opinion articles (n = 1), or being included from another publication (n = 1). This led to 29 publications in the review (the PRISMA flow diagram is available in supplementary materials).

### Descriptive Characteristics of Publications

Table 1 presents an overview of the publications, encompassing 19 distinct CV programs, 89% of which were in the United States, spanning 9 cities and the District of Columbia. A substantial proportion (59%) emerged from 2013 to 2023. The publications included 15 reports (52%), 12 peer-reviewed journal articles (41%), and 2 dissertations (7%). Study designs varied considerably, and 20 studies (69%) measured effectiveness, defined by a primary outcome of changes in fatal or nonfatal violence. Among these, a quarter concluded that the CV program under study was effective or had indicators of effectiveness, half reported mixed results, including decreases in violence in some neighborhoods and no change or increase in violence in other neighborhoods, and another quarter concluded that the CV program did not change or increase violence.

### Characteristics of Implementation Science

Although no publications explicitly mentioned the use of IS, eighteen studies (62%) included IS elements. Among those, 9 (50%) included characteristics of the implementation process,^4,31-35^ 6 (33%) discussed contextual determinants (ie, factors that influenced implementation)^4,8,35-37^ with 1 paper using an IS framework, ^34^ the CFIR.^26^ Furthermore, 3 publications (17%) featured implementation strategies^8,38,39^ and potential mechanisms by which strategies impact outcomes, including attitudes, behaviors, knowledge, and beliefs.^40-42^ The publications examining implementation strategies focused explicitly on conflict mediation and street outreach, and no studies tested strategies against each other. Instead, the authors described the characteristics and variations among the strategies. One paper examined conflict mediation strategies with associated risk factors and their likelihood of resolving conflict.^38^ Finally, among those measuring implementation (n = 18), ten publications (55%) reported on implementation outcomes, with the majority (80%) focusing on fidelity^5,6,10,37,39-44^ (the extent to which the intervention was delivered as planned), followed by one study that examined the feasibility of the local context to implement the model,^45^ cost-effectiveness,^37^ and acceptability of using guns to resolve conflict.^42^ Finally, the majority (77%) of studies had minimal engagement of communities in the study design (eg, decision-making power, assigned specific roles, or informed about events surrounding research activities).^46^

### Factors Influencing Implementation

Among the 6 papers, we identified several facilitators and barriers to implementation,^4,8,35-37^ defined as ‘contextual determinants’^15^ in IS. We summarize these in the following paragraphs, organized by the CFIR domain: 1) intervention characteristics, such as factors related to flexibility, cost, and complexity of the intervention; 2) outer setting, including coordination with partner organizations, external policies, and relationships with other programs; 3) inner setting, including the size and resources among the agencies running the program; 4) individual characteristics, including those related to staff; and 5) processes, including planning and executing strategies.

Factors related to the success of the intervention include those such as community trust, the complexity of a multilevel intervention, sustainable sources of funding and resources, the ability of the program to adapt the intervention to changing situations at the local level, and the relative advantage associated with not working with law enforcement.^44^ For example, relationships with law enforcement were identified as facilitators when interactions occurred with project directors or program staff not directly involved in street outreach. However, the same relationships served as a barrier when fostered through outreach workers because they were seen as compromising the credibility of the outreach worker.^34^ In one study, the programming cost was low compared with other approaches, although programs often faced barriers to sustaining resources.^4^

Relatedly, funding streams were identified as strong determinants within the inner and outer settings. Shifts in funding resulted in reductions in staffing and the inability to carry out all program components.^31,34^ In addition, programs faced the threat of being shut down when they could not demonstrate that their efforts were reducing violence, despite the difficulty of establishing a causal link due to challenges such as finding suitable comparison sites.^47^ Programs benefitted from establishing strong and trustworthy relationships with the community and external organizations, having access to wraparound services for clients (eg, mental health treatment, relocation services), and stable, multiyear funding to retain staff and community resources.^4,8,31,44^ For example, CV programs often face funding challenges, limiting expansion, service provision, and support services such as legal advocacy and job readiness.^10,43,44^

Other determinants were individual characteristics related to program leaders, the host agency, and staff. For example, the decision to hire people formerly incarcerated and/or with lived community violence experience provided a common foundation for dialog and relatability between workers and participants,^31,34^ which helped develop rapport, gain trust, and support positive behavior change. However, barriers arose due to several factors: lack of communication and teamwork among staff; staff history of ‘snitching’ or ongoing involvement in illegal activities (eg, drug selling, gun-related violence); and staff history as offenders involving children or partners. Additionally, staff struggled to adapt to a professional environment (eg, punctuality, following protocols), lacked administrative and mental health support, and faced job-related stressors. These issues led to burnout and turnover among staff, with some tempted to return to their previous lifestyle.^34^

Finally, several determinants related to the process of delivery either strengthened or challenged implementation. These included the visible presence of the program or its staff in the community, hiring and training practices, the consistency and timing of program delivery, the engagement of community stakeholders and providers, the inclusion of team input, the tailoring of mediation techniques by type of violence, the visibility of public information, and the constant use of data and surveillance information to identify neighborhoods and individuals at high risk of committing violence.^5,34,43^ Programs also benefitted from incorporating phased implementation, which allowed sites time to build relationships and partnerships within the community before full implementation and provided an adjustment period for staff who were still learning adjustment professional skills necessary for the job.^45^

### Implementation Strategies

We identified 42 implementation strategies, 14 of which (33%) aligned with the ERIC database.^48^ This resulted in 6 categories: hiring and retention, training, core components of the model—detecting and interrupting violence, identifying and treating high-risk individuals, mobilizing the community to change norms—and general cross-cutting strategies plus maintenance and sustainability. Table 2 (supplementary material) provides the name and definition of each strategy, with examples illustrated in the following text.

Category 1: Hiring and retention were discussed across 5 papers (17%) ^5,34,41,44,49^ and resulted in 5 implementation strategies, including hiring panels, conducting background checks, engaging the team in decision-making, and providing employee support and fair compensation. Hiring panels, background checks, and local consensuses were established to address political pressures associated with individuals previously arrested and some convicted of crimes, confirm the candidates’ credibility in the streets, and ensure that they were not actively involved in criminal activities or had prior offenses in child maltreatment and/or intimate partner violence.^4^ Therapeutic services were provided to address burnout and trauma.

Category 2: Staff training was a key component in 5 (17%) papers,^4,5,32,36,44^ resulting in 6 distinct implementation strategies. This included initial week-long training as the ideal, followed by ongoing training, often monthly and weekly meetings between the staff member and their supervisor, shadowing, and role-playing. The training topics varied and included those related to job functions and those related to professional skills. Some programs also offered training for the broader community. For example, the Aim4Peace (A4P) Violence Prevention Program created a “Community Classroom” on life skills, job readiness, effective parenting, gun violence impact, conflict resolution, and financial management.^38^ Programs often utilize local trainers or consultants from CV Global^2^ to conduct training.^38,45^

Category 3: Detecting and interrupting violence was extracted from more than half (66%) of the papers and focused on 10 discrete strategies during active conflict resolution to keep workers safe, de-escalate conflict, and work toward an agreed-upon solution for all parties. Across all the studies, workers averaged 1 to 3 conflict mediations/month and up to 15 in some programs, depending on team and community size. ^5,8,9,31,32,35,36,37-39,42,43,49-52^

Strategies varied across studies, with common themes around de-escalating or “cooling down” active conflict, addressing concerns of both individuals or groups and following up with both parties. Chicago and Baltimore systematically examined mediation tactics among six focus groups and conceptualized a stepwise approach. This involved “first priorities” of de-escalating active conflict with strategies such as buying time, separating individuals, and attempting to remove weapons. They then determined strategies to structure an intervention, such as bringing people together or calling upon a reliable third party with a closer connection to the individuals. Workers also use specific reasoning and persuasion tactics, often relying on their own stories and talking to individuals about pettiness and harsh consequences if they continue their violent trajectory. Conflicts often ended by bringing the parties to an agreed-upon resolution and having them verbally agree. Workers then followed up with the parties in consecutive weeks.^8^

Similar strategies were observed in other cities, although they were less systematically conceptualized. These included building relationships ahead of time, immediately de-escalating active conflict, and taking steps to resolve/mediate conflicts to avoid future violence. In addition, while not as commonly reported, social media was an emergent strategy. For example, one staff member reported using social media to detect violence among high-risk participants. In this instance, staff members followed their participants identified as high risk on social media to ‘keep tabs’ on any potential conflicts.^34^ Finally, programs tailor their mediation techniques to the situations they respond to in the field, considering factors such as the type of conflict and/or the parties involved.^36^

Category 4: Identifying and treating high-risk individuals includes a set of eight strategies across 12 (41%) papers.^10,33,34,36,37,38,39,40,43,50,53^ Workers typically manage a caseload of 10 to 15 individuals, contacting each person involved in the conflict multiple times during the week, including home visits, and referrals to social service agencies and organizations, which average approximately 10 every month. The programs used criteria to ensure that they were working with individuals at high risk of violence to ensure maximum program impact. The risk criteria included belonging to a gang or being involved in street activity, carrying a weapon, being recently shot or incarcerated, and being close to someone who was shot recently.^10,43^

Staff worked with participants to set and achieve short-, medium-, and long-term goals.^43,44^ In some cases, employment was an important goal to bring in cash and occupy idle time. Outreach workers aided in every step, from writing and completing job applications to attending job fairs and dressing for interviews.^8^ Across publications, the authors highlighted the benefits of following through and providing resources and support services. Support ranged from mentorship to health and social services (eg, obtaining birth certificates, finding housing, attending court hearings, helping pay for prescriptions and medical bills) to lifestyle resources to engage participants in alternatives to criminal activity, such as volunteering, joining a sports team, or obtaining gym membership.^8,36^

Category 5: Changing community norms was classified in more than half of the papers (59%),^5,10,31,32-38,39-42,49-51^ resulting in 6 strategies. Central to success was mobilizing the community, which included a visible presence of the program. Publications described community outreach strategies such as developing and distributing educational messages around “no shooting” or nonviolence, canvassing the community to talk to people and hand out information promoting nonviolence, responding to shootings, and hosting or participating in community events.^5^ Shooting responses typically occur within 48 to 72 h of shootings in targeted communities.^43^ While the exact dosage of community mobilization was not reported in any included documents, consistency in organizing these responses was emphasized across studies. A sentiment repeated in several papers explained how a reliable presence helped ensure that workers were identifiable, that trust was established, and that the community knew that the CV team was dedicated.^44^ Ideally, workers had a daily presence in targeted neighborhoods, averaging reported highs of 300 contacts with individuals from the target community per month. Community events and coalitions brought the community together, encouraged rapport between workers and residents and helped identify individuals who were high risk.^39^

Category 6: Maintenance and Sustainability. Additional overarching implementation strategies (n = 7) were related to program maintenance and sustainability across 13 (45%) papers. Pivotal to these was accessing new funding, with a focus on long-term funding, and creating implementation blueprints or protocols to inform sustainability and replication. Another common theme was establishing strong and trustworthy relationships with external organizations that provided training and services.^39^ For example, collaboration within a broader network of CV programs allows for the sharing of best practices and partnerships with community-based organizations, and in targeted areas, these relationships allow for shared space and resources for programming events.^44^ However, because external relationships and cross-coordination require attention and maintenance, complimentary implementation strategies include phased implementation to allow for relationship development and the practice of obtaining formal commitments from entities such as schools, law enforcement, and hospitals.^4^ Programs such as the Aim4 Peace Violence Prevention Program in Kansas City and Missouri and CV in Chicago, Illinois, implemented formal partnerships with local hospitals to immediately notify program workers when gunshot victims were admitted to emergency rooms, allowing for a quick response, often at the hospital bedside, to prevent retaliations and provide services.^31,32^

### Implementation Research Logic Model

**Figure 1.**
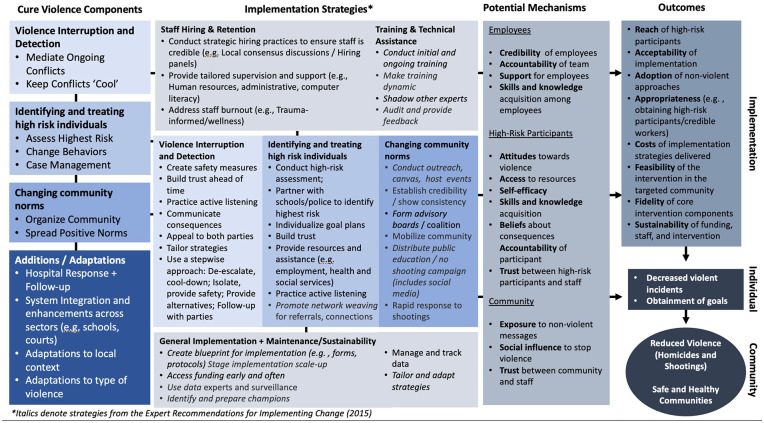
Implementation Research Logic Model. *Italics denote strategies from the Expert Recommendations for Implementing Change (2015).

Figure 1 illustrates an adapted Implementation Research Logic Model (IRLM) that demonstrates the conceptual pathway and implementation process leading to the overall impact of the intervention. The IRLM begins with the core components of the CV initiative, incorporating suggested additions and adaptations.^2^ It then transitions to implementation strategies throughout the lifecycle of the intervention, from pre/early planning to long-term sustainability. This approach allows us to include high systems-level strategies, such as those related to hiring, training, and sustainability, which emerged as key facilitators and barriers to implementation in our review. Next, we attempted to summarize potential mechanisms through which implementation strategies may influence both effectiveness and implementation outcomes. This synthesis drew from existing CV and IS frameworks, ^1,17,18,26^ as well as professional expertise, to construct a comprehensive model.

## Discussion

The aim of this scoping review was to examine the implementation, as opposed to the impact, of CVs. The first objective was to elucidate implementation determinants, strategies, mechanisms and outcomes to build a CV implementation research logic model (IRLM). Publications had a considerable amount of variation in study design and measures of implementation, making comparisons across studies difficult. Rather, the review derived factors that influenced implementation and a related set of 42 implementation strategies from early to late implementation that served as a basis for an adapted IRLM.

Among the 42 implementation strategies listed, 14 (33%) were at least moderately aligned with the ERIC database (Table 2).^48^ Aligning strategies with ERIC supports consistent terminology for research and application. Fewer than half of the strategies matched ERIC, indicating opportunities to expand ERIC's applicability beyond clinical contexts.

Identifying and defining strategies in the IRLM offers distinct opportunities for researchers, practitioners, and policymakers. First, it provides a research framework to test and determine which strategies are most efficient and effective in achieving the desired outcomes. Second, a common framework enables researchers to better identify conceptual linkages between strategies, mechanisms, and outcomes. For practitioners, particularly those in the early stages of implementation, a defined set of strategies serves as an implementation blueprint, facilitating protocol development, staff training, and fidelity in procedures. Practitioners and researchers can also use the proposed IRLM as a tool to adapt and tailor strategies in real-world settings. For example, they can begin by listing strategies and then engage stakeholders to sort and rank them based on their appropriateness or perceived impact. These findings complement recommendations to codevelop theories of change for improved CVI design and evaluation. ^54^

The adapted IRLM provides a tool and novel contribution to the field. Having the IRLM as a community plan to implement a community violence intervention (CVI) can help identify, adapt and tailor implementation strategies. It also provides the research community with a template to plan implementation research studies that can help understand how variations in implementation may impact effectiveness outcomes. Overall, integrating a common framework from the IS into the ecosystem of CVI research establishes a connection with the IS moving forward and provides a foundation for future work.

The second objective of this study was to identify gaps in the IS literature as applied to CV. Although publications included substantial information on the characteristics and factors related to the implementation of CV programs, none of the publications explicitly referred to IS or used consistent implementation terminology or research methods. This makes it difficult to compare implementation across studies, determine which strategies within studies are most effective, or understand how a given context helps or hinders implementation. Understanding the relationships between processes and impacts would have been particularly useful in cities where a community violence (CV) program succeeded in one neighborhood but failed to achieve measurable impact or caused harm in others.

In addition, given the limited publications examining mechanisms and implementation outcomes, we draw on a broader literature review, professional expertise, and a combination of existing frameworks, including the CV Logic Model (2015) and Proctor’s implementation outcomes framework (2011), to populate proposed mechanisms and outcomes within the IRLM. While this approach provides a starting point and addresses a gap in the literature, further research is needed to better delineate mechanisms and their connections to outcomes.

Furthermore, among the common implementation outcomes, only fidelity was consistently reported. While it is important to understand if the CV components are being implemented as planned, there is a wide array of implementation outcomes^20^ that need to be better understood to provide insights into mechanisms and help inform implementation.

Importantly, few studies have employed community-engaged research methods in their study design, representing an opportunity for the field, particularly given the focus of community-centered work. Community-engaged research methods elicit community perspectives and may offer more meaningful benefits than traditional research does.^55^

Finally, given the mixed outcomes (positive, mixed, iatrogenic), there is a need to study implementation systematically to determine if initiatives that do not yield reductions in violence reflect a failure of implementation, the CV intervention, or some combination. Such insights can inform policy development, planning, and efficient use of resources. Furthermore, by naming specific implementation strategies, CVI researchers and practitioners can employ a common language, better implement fidelity, and test and compare strategies to learn, which are the greatest drivers of outcomes.

### Limitations

This study has several limitations. The coding and analysis were conducted by one primary author, potentially introducing subjective biases. To address this, we employed a secondary reviewer, and the coauthors provided oversight. In addition, while the limited number of studies impacts generalizability, it also serves as an indicator that the field is early in its development, and therein lies an opportunity to move forward with greater appreciation and calibration toward the IS. Furthermore, the dynamics of IS in CV programs can vary, and findings from one program may not necessarily be universally applicable.

Additionally, components for the IRLM were extracted from a range of study designs and publication types that did not empirically test or explicitly focus on these implementation components. Consequently, the strategies proposed in the IRLM are presented at face value, limiting their validity and reliability. Moreover, even with the information obtained, we were unable to provide all recommended specifications for strategies, such as dosage. This situation leaves unanswered questions that could impact how practitioners apply the model. Future studies could build upon this review, perhaps systematically assessing the quality of research designs and assessing whether this quality is related to the outcomes.

Finally, the IRLM has an extensive number of strategies to choose from. This review is not able to recommend priority strategies. However, communities can use the strategies listed as a guide for local adaptation and prioritize to the local context. In addition, this provides a platform for researchers to test strategies against each other using rigorous implementation research study designs to determine which ones are most likely to lead to the intended outcomes.

## Conclusion

This paper provides a starting point for incorporating an implementation science (IS) lens in community violence (CV) programming. As the field has mixed results from effectiveness studies, this review offers a valuable resource to guide policymakers, funders, practitioners, communities, and researchers in optimizing the impact of CV and similar interventions. The scoping review advances our understanding of CV implementation but also underscores the vital role of the IS in shaping contextually relevant gun violence prevention initiatives. The adapted IRLM serves as a roadmap to help practitioners in the planning, implementation, and sustainability of CVs and other common CVIs and to inform reproducibility and rigor in research.

## Supplemental Material

sj-docx-1-inq-10.1177_00469580251360956 – Supplemental material for Community-Based Efforts to Reduce Violence: A Scoping Review on the Implementation of Cure Violence Supplemental material, sj-docx-1-inq-10.1177_00469580251360956 for Community-Based Efforts to Reduce Violence: A Scoping Review on the Implementation of Cure Violence by Sara Solomon, Caterina G. Roman, Melissa Davey-Rothwell, Ruth Abaya, Daniel Webster and Shannon Frattorolli in INQUIRY: The Journal of Health Care Organization, Provision, and Financing

sj-pdf-1-inq-10.1177_00469580251360956 – Supplemental material for Community-Based Efforts to Reduce Violence: A Scoping Review on the Implementation of Cure Violence Supplemental material, sj-pdf-1-inq-10.1177_00469580251360956 for Community-Based Efforts to Reduce Violence: A Scoping Review on the Implementation of Cure Violence by Sara Solomon, Caterina G. Roman, Melissa Davey-Rothwell, Ruth Abaya, Daniel Webster and Shannon Frattorolli in INQUIRY: The Journal of Health Care Organization, Provision, and Financing

## References

[1] Butts JA Roman CG Bostwick L Porter JR. Cure Violence: A public health model to reduce gun violence. Annu Rev Public Health. 2015;36(1):39-53. doi:10.1146/annurev-publhealth-031914-122509 25581151

[bibr2-00469580251360956] Cure Violence Global. The Cure Violence approach. Published 2021. Accessed December 14, 2021. https://cvg.org/what-we-do/

[bibr3-00469580251360956] Cure Violence Global. About Cure Violence. Published 2024. Accessed July 13, 2024. https://cvg.org/about/

[bibr4-00469580251360956] Skogan WG Hartnett SM Bump N Dubois J. Evaluation of CeaseFire-Chicago. 2009. Accessed July 13, 2024. http://www.northwestern.edu/ipr/publications/ceasefire.html

[bibr5-00469580251360956] Boyle DJ Lanterman JL Pascarella JE Cheng CC. The impact of Newark’s Operation Ceasefire on trauma center gunshot wound admissions. Justice Res Policy. 2010;12(2):105-123. doi:10.3818/jrp.12.2.2010.105

[bibr6-00469580251360956] Webster DW Whitehill JM Vernick JS Curriero FC. Effects of Baltimore’s Safe Streets program on gun violence: A replication of Chicago’s CeaseFire program. J Urban Health. 2013;90(1):27-40. doi:10.1007/s11524-012-9731-5 22696175 PMC3579298

[bibr7-00469580251360956] Buggs SA Webster DW Crifasi CK. Using synthetic control methodology to estimate effects of a Cure Violence intervention in Baltimore, Maryland. Inj Prev. 2022;28(1):61-67. doi:10.1136/injuryprev-2020-044056 33558396 PMC9019528

[8] Whitehill JM Webster DW Frattaroli S Parker EM. Interrupting violence: How the CeaseFire program prevents imminent gun violence through conflict mediation. J Urban Health. 2014;91(1):84-95. doi:10.1007/s11524-013-9796-9 23440488 PMC3907621

[bibr9-00469580251360956] Wilson JM Chermak SM McGarrell EF , et al. Community-Based Violence Prevention: An Assessment of Pittsburgh’s One Vision, One Life Program. RAND Corporation; 2010.

[bibr10-00469580251360956] Fox AM Katz CM Choate DE Hedberg EC. Evaluation of the Phoenix TRUCE Project: A replication of Chicago CeaseFire. Justice Q. 2015;32(1):85-115. doi:10.1080/07418825.2014.902092

[bibr11-00469580251360956] Hureau DM Braga AA Lloyd T Winship C. Streetwork at the crossroads: An evaluation of a street gang outreach intervention and holistic appraisal of the research evidence. Criminology. 2023;61(4):758-794. doi:10.1111/1745-9125.12353

[bibr12-00469580251360956] Pugliese K Odér P Hudson T Butts J. Community Violence Intervention at the Roots (CVI-R): Building Evidence for Grassroots Community Violence Prevention. 2022.

[bibr13-00469580251360956] Glasgow RE Chambers D. Developing robust, sustainable, implementation systems using rigorous, rapid and relevant science. Clin Transl Sci. 2012;5(1):48-55. doi:10.1111/j.1752-8062.2011.00383.x 22376257 PMC5439908

[bibr14-00469580251360956] Bauer MS Damschroder L Hagedorn H Smith J Kilbourne AM. An introduction to implementation science for the nonspecialist. BMC Psychol. 2015;3(1):32. doi:10.1186/s40359-015-0089-9 26376626 PMC4573926

[bibr15-00469580251360956] Nilsen P Bernhardsson S. Context matters in implementation science: A scoping review of determinant frameworks that describe contextual determinants for implementation outcomes. BMC Health Serv Res. 2019;19(1):189. doi:10.1186/s12913-019-4015-3 30909897 PMC6432749

[bibr16-00469580251360956] Curran GM. Implementation science made too simple: a teaching tool. Implement Sci Commun. 2020;1(1):27. doi:10.1186/s43058-020-00001-z 32885186 PMC7427844

[bibr17-00469580251360956] Proctor EK Powell BJ McMillen JC. Implementation strategies: recommendations for specifying and reporting. Implement Sci. 2013;8(1):139. doi:10.1186/1748-5908-8-139 24289295 PMC3882890

[bibr18-00469580251360956] Smith JD Li DH Rafferty MR. The implementation research logic model: a method for planning, executing, reporting, and synthesizing implementation projects. medRxiv. 2020:1-12. doi:10.1101/2020.04.05.20054379 PMC7523057 32988389

[bibr19-00469580251360956] Freedman AM Simmons S Lloyd LM , et al. Public Health Training Center evaluation. Health Promot Pract. 2014;15(1_suppl):80S-88S. doi:10.1177/1524839913509271 24578370

[bibr20-00469580251360956] Proctor E Silmere H Raghavan R , et al. Outcomes for implementation research: conceptual distinctions, measurement challenges, and research agenda. Adm Policy Ment Health. 2011;38(2):65-76. doi:10.1007/s10488-010-0319-7 20957426 PMC3068522

[bibr21-00469580251360956] Mak S Thomas A. Steps for conducting a scoping review. J Grad Med Educ. 2022;14(5):565-567. doi:10.4300/JGME-D-22-00621.1 36274762 PMC9580325

[bibr22-00469580251360956] Tricco AC Lillie E Zarin W , et al. PRISMA extension for scoping reviews (PRISMA-ScR): checklist and explanation. Ann Intern Med. 2018;169(7):467-473. doi:10.7326/M18-0850 30178033

[23] Center for Open Science. OSF Home. Accessed December 4, 2024. https://www.osf.io

[24] Penn Libraries. Covidence. Accessed April 22, 2023. https://guides.library.upenn.edu/covidence

[25] Vorkoper S Tahlil KM Sam-Agudu NA , et al. Implementation science for the prevention and treatment of HIV among adolescents and young adults in Sub-Saharan Africa: a scoping review. AIDS Behav. 2023;27(S1):7-23. doi:10.1007/s10461-022-03770-x 35947233 PMC10191963

[26] Damschroder LJ Reardon CM Opra Widerquist MA Lowery J. Conceptualizing outcomes for use with the Consolidated Framework for Implementation Research (CFIR): the CFIR Outcomes Addendum. Implement Sci. 2022;17(1):7. doi:10.1186/s13012-021-01181-5 35065675 PMC8783408

[bibr27-00469580251360956] Kemp CG Wagenaar BH Haroz EE. Expanding hybrid studies for implementation research: intervention, implementation strategy, and context. Front Public Health. 2019;7:325. doi:10.3389/fpubh.2019.00325 31781528 PMC6857476

[bibr28-00469580251360956] Vejnoska SF Mettert K Lewis CC. Mechanisms of implementation: an appraisal of causal pathways presented at the 5th biennial Society for Implementation Research Collaboration (SIRC) conference. Implement Res Pract. 2022;3:263348952210862. doi:10.1177/26334895221086271 PMC9924245 37091081

[29] Waltz TJ Powell BJ Fernández ME Abadie B Damschroder LJ. Choosing implementation strategies to address contextual barriers: diversity in recommendations and future directions. Implement Sci. 2019;14(1):42. doi:10.1186/s13012-019-0892-4 31036028 PMC6489173

[30] Ramanadhan S Revette AC Lee RM Aveling EL. Pragmatic approaches to analyzing qualitative data for implementation science: an introduction. Implement Sci Commun. 2021;2(1):70. doi:10.1186/s43058-021-00174-1 34187595 PMC8243847

[31] Watson-Thompson J Harsin J Stewart D Everett M Esiaka C. AIM4PEACE Evaluation Report Summary, 2018–2020. Lawrence, KS: Center for Community Health and Development, University of Kansas; 2022.

[bibr32-00469580251360956] Slutkin G Ransford C Decker B Volker K. Cure Violence – An Evidence-Based Method to Reduce Shootings and Killings. Chicago, IL: Cure Violence; 2014.

[bibr33-00469580251360956] Cure Violence Global. 2016 Progress Report. 2016. Accessed July 13, 2024. https://cvg.org/wp-content/uploads/2019/09/NOLAFORLIFE_ProgressReport_2016_LONG_070816-web-1.pdf

[bibr34-00469580251360956] Defries Gallagher A . A Qualitative Analysis of the Recommended Implementation and Replication of the Cure Violence Model According to New York City and Chicago Program Staff Interviews. New York, NY: City University of New York (CUNY); 2021.

[bibr35-00469580251360956] Adams EB Maguire ER. Qualitative evidence on the implementation of Cure Violence in Trinidad and Tobago. Prev Sci. 2023;24(4):774-784. doi:10.1007/s11121-023-01500-w 36729350

[bibr36-00469580251360956] Dymnicki AB Henry D Quintana E Wisnieski E Kane C. Outreach workers’ perceptions of positive and negative critical incidents: Characteristics associated with successful and unsuccessful violence interruption. J Community Psychol. 2013;41(2):200-217. doi:10.1002/jcop.21523

[bibr37-00469580251360956] Maguire E Oakley M Corsaro N. Evaluating Cure Violence in Trinidad and Tobago. 2018. doi:10.18235/0001427

[bibr38-00469580251360956] Stewart D Jessop N Watson-Thompson J. Examining conflict mediation to prevent violence through multisector partnerships. Peace Confl. 2021;27(2):170-181. doi:10.1037/pac0000536

[39] Whitehill JM. Street Outreach for Youth Violence Prevention: Lessons from Implementation of the CeaseFire Model in Chicago and Baltimore. Johns Hopkins University; 2011.

[bibr40-00469580251360956] Gorman-Smith D Cosey-Gay F. Residents and Clients’ Perceptions of Safety and CeaseFire Impact on Neighborhood Crime and Violence. University of Chicago, School of Social Service Administration; 2006.

[bibr41-00469580251360956] Butts JA John C College J Wolff KT Misshula E Delgado SA. Effectiveness of the Cure Violence Model in New York City. 2012. Accessed September 1, 2023. https://academicworks.cuny.edu

[bibr42-00469580251360956] Milam AJ Furr-Holden CD Leaf P Webster D. Managing conflicts in urban communities: Youth attitudes regarding gun violence. J Interpers Violence. 2016;33(24):3815-3828. doi:10.1177/0886260516639584 27021734 PMC8626823

[bibr43-00469580251360956] Picard-Fritsche S Cerniglia L. An Evaluation of Crown Heights Save Our Streets, a Replication of the Cure Violence Model. Center for Court Innovation; 2013.

[44] Delgado SA Alsabahi L Wolff K Butts J. The Effects of Cure Violence in the South Bronx and East New York, Brooklyn. 2017. Accessed April 15, 2023. https://johnjayrec.nyc/2017/10/02/cvinsobronxeastny/

[45] Ransford C. Cure Violence Internal Report. Cure Violence; 2016.

[46] Gibbons GH Pérez-Stable EJ. Harnessing the power of community-engaged research. Am J Public Health. 2024;114(S1):S7-S11. doi:10.2105/AJPH.2023.307528 PMC10785163 38207272

[47] Buggs S. Community-Based Violence Interruption and Public Safety. 2022. Accessed April 15, 2023. https://craftmediabucket.s3.amazonaws.com/uploads/AVCJIReport_Community-BasedViolenceInterruptionPublicSafety_Buggs_v2.pdf

[48] Powell BJ Waltz TJ Chinman MJ , et al. A refined compilation of implementation strategies: results from the Expert Recommendations for Implementing Change (ERIC) project. Implement Sci. 2015;10(1):21. doi:10.1186/s13012-015-0209-1 25889199 PMC4328074

[49] Chwalisz N. Beating the gun—One conversation at a time? Evaluating the impact of DC’s “Cure the Streets” public health intervention against gun violence. Crime Delinq. 2023; 71(6-7), 1906-1928. doi:10.1177/00111287231160735

[bibr50-00469580251360956] City of New Orleans. Nola for Life: 2016 Progress Report. 2016. Accessed November Roman C, Reeves K, Bellamy M. *Philadelphia CeaseFire: Findings from the Impact Evaluation*. 2017. Accessed April 15, 2023. https://cvg.org/wp-content/uploads/2020/03/SummaryofPhilaCeaseFireFindingsFormatted_Jan2017.pdf

[bibr51-00469580251360956] Webster DW Buggs SA Crifasi CK. Estimating the Effects of Efforts to Reduce Gun Violence Johns Hopkins Bloomberg School of Public Health, Center for Gun Policy and Research; 2018.

[52] Whitehill JM Parker EM Mendel J Webster DW Vernick JS. Evaluation of Baltimore’s Safe Streets Program: Effects on Attitudes, Participants’ Experiences, and Gun Violence. 2015.

[53] Skogan WG Hartnett SM Bump N Dubois J. Executive Summary: Evaluation of CeaseFire-Chicago. 2009.

[54] Schleimer JP Lyons VH Smith D , et al. Codeveloping theories of change for improved community-based violence intervention evaluation. J Trauma Acute Care Surg. 2024;97(2):278-285. doi:10.1097/TA.0000000000004277 38509040

[55] Wallerstein N Duran B. Community-based participatory research contributions to intervention research: The intersection of science and practice to improve health equity. Am J Public Health. 2010;100(Suppl 1):S40-S46. doi:10.2105/AJPH.2009.184036 PMC2837458 20147663

