# Supplementary material for: Community-Based Efforts to Reduce Violence: A Scoping Review on the Implementation of Cure Violence
Source: Inquiry. 2025 Jul 28;62:00469580251360956. doi: 10.1177/00469580251360956 (PMC12304640; doi:10.1177/00469580251360956)
Supplement: sj-docx-1-inq-10.1177_00469580251360956 – Supplemental material for Community-Based Efforts to Reduce Violence: A Scoping Review on the Implementation of Cure Violence [file sj-docx-1-inq-10.1177_00469580251360956.docx]

**Figure 1.**PRISMA Table

Studies from databases/registers **(n = 123)**

Google Scholar (n = 48)

Catalyst Library JHU (n = 22)

PubMed (n = 21)

Citation searching (n = 14)

Cure Violence Website (n = 5)

Unspecified (n = 13)

Studies included in review **(n = 29)**

Studies excluded **(n = 46)**

Studies not retrieved **(n = 0)**

Studies assessed for eligibility **(n = 48)**

Studies sought for retrieval **(n = 48)**

Studies screened **(n = 94)**

Studies excluded **(n = 19)**

Opinion (n = 1)

Redundant (n = 1)

Conference Proceedings (n = 4)

Not a process or impact evaluation (n = 3)

Not explicit focus on Cure Violence (n = 10)

**Identification**

References removed **(n = 29)**

Duplicates identified manually (n = 0)

Duplicates identified by Covidence (n = 29)

Marked as ineligible by automation tools (n = 0)

**Screening**

**Included**
